# Cell signaling during *Trypanosoma cruzi* invasion

**DOI:** 10.3389/fimmu.2012.00361

**Published:** 2012-11-28

**Authors:** Fernando Y. Maeda, Cristian Cortez, Nobuko Yoshida

**Affiliations:** Departamento de Microbiologia, Imunologia e Parasitologia, Escola Paulista de Medicina, Universidade Federal de São PauloSão Paulo, São Paulo, Brazil

**Keywords:** *Trypanosoma cruzi*, cell invasion, cell signaling, Ca^2^^+^ mobilization, metacyclic rypomastigote, tissue culture trypomastigote

## Abstract

Cell signaling is an essential requirement for mammalian cell invasion by *Trypanosoma cruzi*. Depending on the parasite strain and the parasite developmental form, distinct signaling pathways may be induced. In this short review, we focus on the data coming from studies with metacyclic trypomastigotes (MT) generated *in vitro* and tissue culture-derived trypomastigotes (TCT), used as counterparts of insect-borne and bloodstream parasites, respectively. During invasion of host cells by MT or TCT, intracellular Ca^2^^+^ mobilization and host cell lysosomal exocytosis are triggered. Invasion mediated by MT surface molecule gp82 requires the activation of mammalian target of rapamycin (mTOR), phosphatidylinositol 3-kinase (PI3K), and protein kinase C (PKC) in the host cell, associated with Ca^2^^+^-dependent disruption of the actin cytoskeleton. In MT, protein tyrosine kinase, PI3K, phospholipase C, and PKC appear to be activated. TCT invasion, on the other hand, does not rely on mTOR activation, rather on target cell PI3K, and may involve the host cell autophagy for parasite internalization. Enzymes, such as oligopeptidase B and the major *T. cruzi* cysteine proteinase cruzipain, have been shown to generate molecules that induce target cell Ca^2^^+^ signal. In addition, TCT may trigger host cell responses mediated by transforming growth factor β receptor or integrin family member. Further investigations are needed for a more complete and detailed picture of *T. cruzi* invasion.

## INTRODUCTION

The hallmark of host cell invasion by *Trypanosoma cruzi*, a process that involves diverse parasite and host cell components, is the activation of signal transduction pathways leading to elevation in cytosolic Ca^2+^ concentration in both cells (Docampo and Moreno, 1996; Burleigh and Andrews, 1998; Yoshida, 2006). Ca^2+^-dependent disruption of host cell actin cytoskeleton that follows interaction with *T. cruzi* facilitates the mobilization of lysosomes to the cell periphery, where the fusion with the plasma membrane contributes for the biogenesis of parasitophorous vacuole, inhibition of this event resulting in impaired parasite internalization (Tardieux et al., 1992; Rodriguez et al., 1995; Martins et al., 2011).

Studies with metacyclic trypomastigotes (MT) generated *in vitro* and tissue culture-derived trypomastigotes (TCT), used as counterparts of insect-borne and bloodstream parasites, respectively, have disclosed that these developmental forms engage distinct sets of molecules and diverse strategies to induce host cell Ca^2+^ signaling and lysosomal exocytosis required for their internalization. Here we summarize the data from experiments performed mostly with non-phagocytic mammalian cells, aiming at understanding the signaling events that lead to *T. cruzi* invasion.

## MT SURFACE MOLECULES THAT TRIGGER HOST CELL SIGNALING DURING INVASION

Adhesion to host cells is the first step for *T. cruzi *invasion. Surface glycoproteins with cell adhesion properties expressed in MT, such as gp90, gp82, gp30, and gp35/50, which are differentially expressed in different strains, bind to target cells in a receptor-mediated manner and trigger signaling pathways that may result or not in efficient parasite internalization (Yoshida, 2006).

**FIGURE 1:**
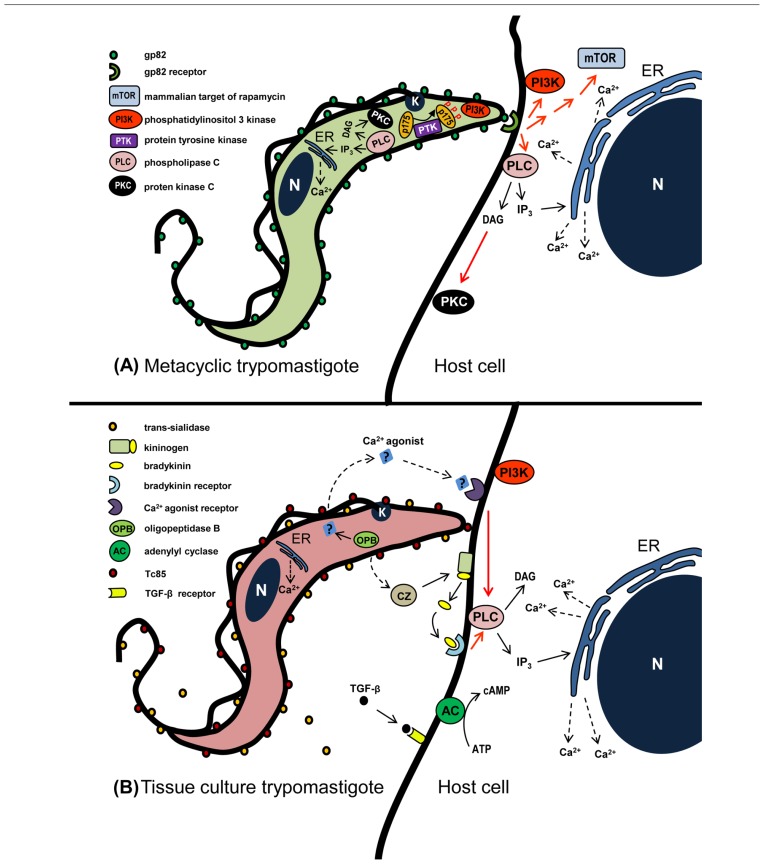
**Schematic representation of signaling molecules and pathways that may be activated during *T. cruzi* entry into non-phagocytic mammalian cells**. **(A)** In metacyclic forms that enter host cells in gp82-mediated manner, activation of PLC generates DAG and IP_3_. DAG stimulates PKC and IP_3_ promotes Ca^2+^ release from IP_3_-sensitive compartments. PI3K and PTK are also activated, the latter mediates phosphorylation of p175. In the host cell, the recognition of gp82 by its receptor triggers the activation of PI3K, mTOR, and PLC, the latter generating DAG and IP_3_. DAG stimulates PKC and IP_3_ promotes Ca^2+^ release from endoplasmic reticulum (ER). **(B)** During TCT interaction with host cells, a Ca^2+^ agonist generated by parasite OPB binds to its receptor and triggers PLC activation. Then IP_3_-mediated Ca^2+^ release from ER ensues. Bradykinin, produced from kininogen by the action of TCT cruzipain, binds to bradykinin receptor and triggers PLC activation. Red arrows indicate activation, possibly not directly, but through as one or more as yet undefined elements.

Gp82, identified by the monoclonal antibody (mAb) 3F6, is a MT-specific surface molecule (Teixeira and Yoshida, 1986). It is a member of a multigene family that belongs to the gp85/*trans*-sialidase superfamily (Araya et al., 1994). Several pieces of evidence indicate that gp82 is engaged by highly infective *T. cruzi* strains to enter host cells (Ramirez et al., 1993; Cortez et al., 2012a). Gp82 is conserved among *T. cruzi* strains from divergent genetic groups, displaying >90% peptide sequence identity (Maeda et al., 2011). MT invasion mediated by gp82 triggers the target cell signaling cascades that result in cytosolic Ca^2+^ mobilization, an event detectable in mammalian cells susceptible to *T. cruzi* infection, such as HeLa and Vero cells, but not in *T. cruzi*-resistant K562 cells (Ruiz et al., 1998). Following gp82 recognition by its still undefined receptor, the available data indicate that Ca^2+^ is released from thapsigargin-sensitive stores, independent of inositol 1,4,5-triphosphate (IP_3_), or upon activation of phospholipase C (PLC), generating diacylglycerol (DAG) and IP_3_, the former activates protein kinase C (PKC) and the latter promotes Ca^2+^ release from IP_3_-sensitive compartments such as endoplasmic reticulum (ER; Ferreira et al., 2006; Maeda et al., 2012; **Figure 1A**). In addition to PKC, two other kinases participate in gp82-mediated MT invasion, namely the mammalian target of rapamycin (mTOR), a conserved Ser/Thr kinase that regulates diverse cell processes, and the lipid kinase phosphatidylinositol 3-kinase (PI3K), as suggests the diminished parasite invasion of cells pretreated with specific inhibitors of these enzymes (Martins et al., 2011; **Figure 1A**). What are the connections between these kinases can only be inferred at this point from data available in other systems. PI3K may act on mTOR signaling, provided that the phosphorylation of downstream effectors of mTOR, such as S6K1 and 4E-BP1, is sensitive to rapamycin and also to PI3K inhibitor wortmannin (Chung et al., 1994; Mèndez et al., 1996; Hay and Sonenberg, 2004). Another possible functional association is between PKC and mTOR. A pathway linking epidermal growth factor receptor to mTOR that was critically dependent on PKC has been described in glioma (Fan et al., 2009) and the association of a mTOR homolog with PKC has been demonstrated in *Saccharomyces cerevisiae* (Kumar et al., 2000). Under some circumstances, PI3K is activated upstream of PKC (Tong et al., 2000). Phosphatidylinositol-3,4,5-P_3_, a product of PI3K, appears to directly initiate cellular motility via PKC activation (Derman et al., 1997).

**FIGURE 2:**
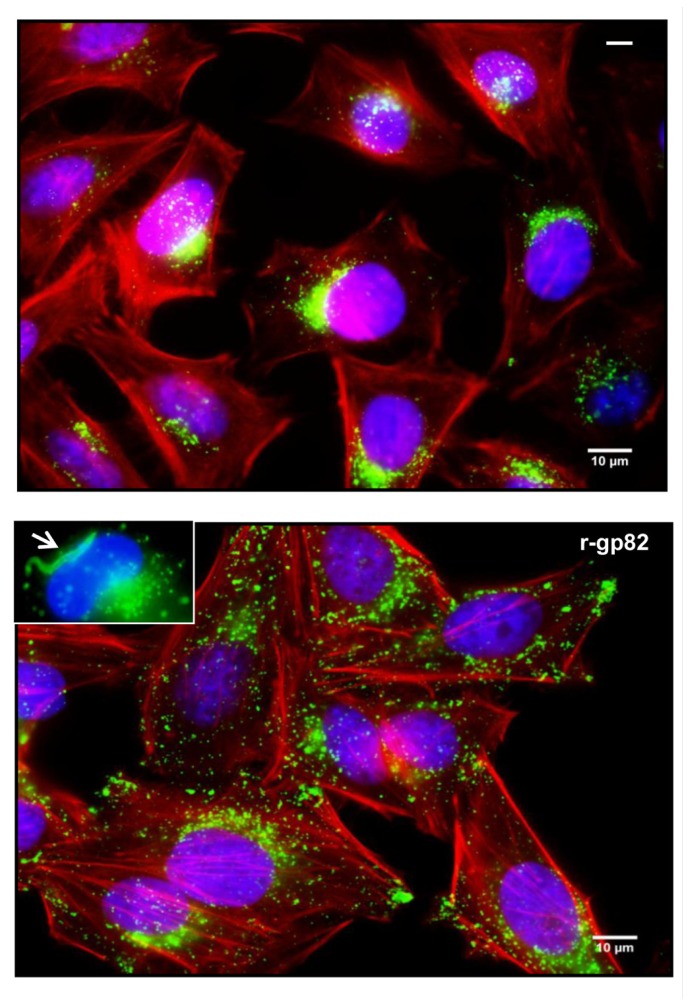
**Lysosome mobilization induced by MT gp82**. Shown are HeLa cells incubated for 1 h in absence or in the presence of the recombinant protein (r-gp82) containing the complete gp82 sequence. Spread of lysosomes (green) from the perinuclear region to the cell periphery is induced by r-gp82. In the inset, recently internalized MT (arrow) inside the parasitophorous vacuole with lysosome marker is shown.

The elevation of cytosolic Ca^2+^ concentration induced by gp82 promotes two associated events that facilitate MT invasion, namely the Ca^2+^-dependent actin cytoskeleton disruption and lysosome mobilization that culminates in exocytosis (Cortez et al., 2006; Martins et al., 2011; **Figure 2**). During gp82-mediated MT invasion, recently internalized parasites are seen within vacuoles that incorporated lysosome markers (**Figure 2**).

Gp30, a MT-specific surface molecule recognized by mAb 3F6 and expressed in gp82-deficient *T. cruzi* strains, is also implicated in cell invasion (Cortez et al., 2003, 2012a). Like gp82, binding of gp30 to target cells induces Ca^2+^ response and lysosome exocytosis, presumably through activation of signaling pathways involving PI3K, mTOR, and PKC (Cortez et al., 2003, 2012a). Gp30 and gp82 are possibly recognized by the same receptor, as suggests the inhibition of host cell entry of both gp30- and gp82-expressing MT by mAb 3F6, as well as by recombinant proteins based on gp30 or gp82 (Cortez et al., 2003, 2012a).

In addition to gp82 or gp30, MT of different *T. cruzi* strains express variable levels of distinct isoforms of a stage-specific surface molecule gp90, which functions as a negative regulator of parasite infectivity (Málaga and Yoshida, 2001). Expression of gp90 at high levels is invariably associated with reduced capacity to enter target cells (Yoshida, 2006). As opposed to gp82 or gp30, and consistent with its role as down modulator of MT invasion, gp90 does not trigger Ca^2+^ signal upon binding to host cells (Ruiz et al., 1998).

Metacyclic trypomastigotes surface molecules gp35/50 recognized by mAb 10D8, expressed in poorly infective *T. cruzi* strains, are highly glycosylated mucin-like glycoproteins enriched in sialic acid and galactose residues that interact with target cells through their carbohydrate portion (Yoshida et al., 1989; Mortara et al., 1992; Schenkman et al., 1993b). Binding of gp35/50 to target cells triggers intracellular Ca^2+^ elevation, but to a lower degree than gp82 (Ruiz et al., 1998). Removal of sialic acid from gp35/50 increases the capacity to trigger target cell Ca^2+^ response and potentiates MT invasion (Yoshida et al., 1997). It appears therefore that sialyl residues impair parasite–host cell interaction and this is in contrast with the findings with TCT (Schenkman et al., 1991). Gp35/50-mediated invasion apparently requires F-actin recruitment, an event that may be associated with activation of adenylyl cyclase that generates cAMP (Ferreira et al., 2006).

The role played by MT secreted components in parasite internalization remains to be investigated. One such component, SAP (serine-, alanine-, and proline-rich protein), which binds to target cells in a receptor-dependent manner and induces Ca^2+^ signal, participates in the gp82-mediated internalization of MT but plays no role in gp35/50-mediated invasion (Baida et al., 2006). It is possible that SAP acts synergistically with gp82, by triggering Ca^2+^ signal that adds to the response induced by gp82.

## SIGNALING PATHWAYS ACTIVATED IN MT DURING INVASION

Gp82-mediated invasion of host cells by MT triggers Ca^2+^ mobilization in the parasite, through signaling cascades involving PLC activation, generation of DAG and IP_3_, leading to Ca^2+^ release from IP_3_-sensitive reservoirs and PKC stimulation (Yoshida et al., 2000; **Figure 1A**). In addition to involvement of PI3K (Maeda et al., 2012), a protein tyrosine kinase (PTK) activation results in phosphorylation of p175, a protein undetectable in non-infective epimastigotes (Favoreto et al., 1998; **Figure 1A**). PTK activation and Ca^2+^ response are possibly associated events, provided that they are both affected by genistein (Yoshida et al., 2000), a PTK inhibitor that reduces MT infectivity (Neira et al., 2002). MT that invade host cells in a gp35/50-mediated manner may require cAMP and acidocalcisomes, the vacuoles containing a Ca^2+^/H^+^ exchange system (Docampo et al., 1995), appear to be the main source of Ca^2+^ required for parasite internalization (Neira et al., 2002).

## TCT-INDUCED SIGNALING EVENTS IN TARGET CELLS

Diverse *T. cruzi* molecules, either secreted and/or expressed on the cell surface, have been implicated in TCT internalization. Among those known to have cell signal-inducing properties are cruzipain, *trans*-sialidase, trypomastigote small surface antigen (TSSA), and a soluble factor of undefined structure.

Cruzipain, the major *T. cruzi* cysteine proteinase expressed in all developmental forms of different strains (Murta et al., 1990; Paiva et al., 1998), participates in TCT internalization and in intracellular parasite development (Meirelles et al., 1992). From experiments using human umbilical vein endothelial cells or CHO cells overexpressing B_2_ type of bradykinin receptor (B_2_R), it was postulated that cruzipain acts on cell-bound kininogen and generates bradykinin that, upon recognition by B_2_R triggers IP_3_-mediated Ca^2+^ influx (Scharfstein et al., 2000; **Figure 1B**), thus promoting parasite invasion, a mechanism that is not ubiquitous, its activation depending on the cell type and the parasite isolate used. Higher expression of functional cruzipain does not correlate with parasite infectivity (Paiva et al., 1998).

*Trypanosoma cruzi* *trans*-sialidase (TS), an enzyme that specifically transfers alpha (2-3)-linked sialic acid from host-derived macromolecules to parasite surface molecules, facilitates TCT invasion by sialylating a TCT-specific epitope Ssp-3, which is recognized by target cells through its sialic acid residues and whose signaling properties are unknown (Schenkman et al., 1991). TS may function as a TCT ligand to host cell alpha 2,3-sialyl receptors as a prelude to invasion (Ming et al., 1993). Signaling activities of TS toward mammalian cells include activation of PI3K/Akt pathway that contributes for survival of Schwann cells (Chuenkova et al., 2001), of mitogen-activated protein kinase (MAPK) or extracellular regulated kinase (ERK) pathways that induce neurite outgrowth in PC12 cells (Chuenkova and Pereira, 2001). TCT binds to TrkA, a receptor tyrosine kinase activated primarily by nerve growth factor, in a manner mediated by TS, inducing TrkA autophosphorylation and PI3K/Akt kinase signaling through TrkA-dependent mechanisms (Chuenkova and PereiraPerrin, 2004). Whether these TS-induced signaling mechanisms are associated with TCT invasion is not known. The participation of host cell sialic acid in TCT invasion has been inferred using Chinese hamster ovary cell mutant that is much less susceptible to infection than the parental cell line (Ciavaglia et al., 1993; Ming et al., 1993; Schenkman et al., 1993a). In macrophages, removal of sialic acid with neuraminidase or its blockage with cationized ferritin increased TCT uptake (Ara'u, 1984; Meirelles et al., 1984).

Recently, Cánepa et al. (2012) reported that peptides based on TSSA, a mucin-like molecule rich in serine and threonine predicted to be *O*-glycosylated (Di Noia et al., 2002), bind to mammalian cells and induce Ca^2+^ signaling. The question whether the native glycosylated TSSA and synthetic TSSA peptides share the same cell adhesion and signaling properties has not been addressed.

A secreted TCT factor of unknown structure has been claimed to trigger host cell Ca^2+^ mobilization in IP_3_-mediated manner (Rodriguez et al., 1995). According to Burleigh et al. (1997), the soluble TCT factor is produced by the action of cytosolic oligopeptidase B (OPB), an enzyme closely related to members of the prolyl oligopeptidase family of serine endopeptidases. The Ca^2+^ agonist, generated from a precursor molecule in TCT cytoplasm, would be exported and its recognition by a target cell receptor, followed by PLC activation and IP_3_ production, would release Ca^2+^ from ER (Caler et al., 1998; **Figure 1B**). OPB null TCT had a diminished cell invasion capacity, a Ca^2+^ signal-inducing activity of low intensity and recruited lysosome in a significantly delayed fashion, but preserved the property to induce cAMP elevation in host cells (Caler et al., 2000), which is associated with the ability to potentiate Ca^2+^-regulated lysosomal exocytosis (Rodriguez et al., 1999). While the ability of Ca^2+^ agonist produced by OPB in disrupting F-actin filaments is associated with increased TCT invasion (Rodriguez et al., 1995), there are reports indicating that actin cytoskeleton disruption results in diminished TCT entry into different cell types, including heart muscle cells (Meirelles et al., 1999; Rosestolato et al., 2002).

Several TCT surface molecules with affinity for extracellular matrix have been implicated in host cell invasion, but little is known about their signal-inducing properties. Among such molecules is a laminin-binding glycoprotein encoded by a multigene Tc85 family belonging to the gp85/*trans*-sialidase superfamily (Giordano et al., 1999). Conserved in all members of gp85/*trans*-sialidase glycoprotein family is the FLY domain (VTVXNVFLYNR). Peptide based on FLY binds to cytokeratin 18 (CK18) on the surface of LLC-MK(2) epithelial cells and promotes dephosphorylation and CK18 reorganization, activating ERK1/2 signaling pathway that leads to increased TCT internalization (Magdesian et al., 2001). This finding with peptide FLY is unlikely to bear any association with TCT entry into host cells because FLY domain is almost completely buried (Cortez et al., 2012b), therefore unavailable for interaction with CK18. In support of this view, transient silencing of CK18 gene in RNAi-treated HeLa cells did not affect binding and invasion of TCT (Claser et al., 2008). Furthermore, a recombinant protein based on amastigote surface protein-2 containing FLY domain failed to bind CK18 (Claser et al., 2008), consistent with the fact that FLY domain is not exposed on the surface.

Cell signaling events during TCT internalization, without association with specific *T. cruzi* molecules, have been reported by many authors. In different cell types, activation of PI3K emerges as a common feature for TCT invasion process. PI3K activated by TCT facilitates lysosome-dependent parasite entry into non-pahgocytic cells (Woolsey et al., 2003). In target cells invaded by a significant fraction of TCT through an lysosome-independent pathway, there is the formation of a host cell plasma membrane-derived vacuole enriched in the lipid products of class I PI3 kinases, initially devoid of lysosomal markers and gradually acquiring lysosome associated membrane protein 1 (Woolsey et al., 2003). This lysosome-independent early event is compatible with the finding that the newly forming TCT compartments first interact with an early endosome and subsequently with other late endosomes, before interaction with lysosomes (Wilkowsky et al., 2002). Using blood trypomastigotes and macrophages, Todorov et al. (2000) found that class I and class III PI3-kinase activities are involved in parasite internalization. PI3K recruitment and assembly of actin filaments were detected at the site of TCT interaction with macrophages (Vieira et al., 2002). In non-phagocytic Vero, L_6_E_9_ and NIH 3T3 cells, as well as in human and J774 murine macrophages, PI3K inactivation was ascertained using specific PI3K inhibitors (Wilkowsky et al., 2001). Concomitant with PI3K activation, a strong activation of protein kinase B (PKB/Akt) occurs and, accordingly, transiently transfected cells containing an inactive mutant PKB are more resistant to infection by TCT as compared to the active mutant-transfected cells (Wilkowsky et al., 2001).

Tissue culture-derived trypomastigotes invasion of macrophages also requires PTKs (Vieira et al., 1994). Tyrosine-phosphorylated residues accumulate at the site of TCT association with the cell surface, co-localizing with macrophage F-actin-rich domains (Vieira et al., 2002). Activation of macrophage PKC induced by recombinant gp83, a TCT surface ligand, was also reported (Villalta et al., 1999). Protein phosphatases may also play a role in TCT internalization. Tyrosine dephosphorylation of several proteins is induced by TCT in L_6_E_9_ myoblasts and the cells, either treated with protein tyrosine phosphatase inhibitors or in the presence of excess phosphotyrosine, become more resistant to invasion by TCT (Zhong et al., 1998). The involvement of alkaline phosphatase has been deduced from experiments with human HEp2 tumor cells that, upon inhibition of the enzyme activity, exhibited a different pattern of actin organization and reduced susceptibility to TCT invasion (Sartori et al., 2003).

Several other host cell components have been implicated in TCT invasion. Ming et al. (1995) found that TCT induce a transforming growth factor β (TGF-β)-responsive reporter gene in TGF-β-sensitive cell lines, and epithelial cells lacking TGF-β receptor I or II, or with dysfunction of the intracellular signaling cascade due to constitutive expression of the cyclin-dependent kinase cdk4 or of the oncogene H-Ras, were more refractory to penetration by TCT. In experiments with human coronary artery smooth muscle cells expressing galectin-3, which increases K-Ras activation and triggers a Ras signal (Elad-Sfadia et al., 2004), there was a decreased TCT adhesion to cells with reduced expression of galectin-3, which was restored by exogenous galectin-3 (Kleshchenko et al., 2004). The β1 subunit of VLA integrin family that links the extracellular matrix to the cortical cytoskeleton was reported to be involved in TCT entry into human macrophages (Fernandez et al., 1993). Recently, acid sphingomyelinase (ASM) was claimed to be required for TCT invasion. Inhibition or depletion of lysosomal ASM markedly reduced the target cell susceptibility to TCT invasion, whereas extracellular addition of ASM stimulated endocytosis, enhanced parasite entry, and restored normal invasion levels in ASM-depleted cells, and ceramide, the product of sphingomyelin hydrolysis, was detected in newly formed parasitophorous vacuoles containing TCT (Fernandes et al., 2011). Triggering of autophagy was also associated with TCT internalization. Romano et al. (2009) have shown that treatment of host cells with mTOR inhibitor rapamycin increased lysosomal-dependent TCT invasion by inducing autophagy. This finding is in sharp contrast with gp82-mediated MT invasion that is inhibited by rapamycin (Martins et al., 2011). Also contrasting with gp82-mediated MT internalization that is impaired in cells prestarved for a short time (Martins et al., 2011), condition that triggers the autophagic pathway, TCT invasion increased upon prestarvation of target cells (Romano et al., 2009). In addition, the absence of Atg5 or the reduced expression of Beclin 1, proteins required at the initial steps of autophagosome formation, reduced the association of parasitophorous vacuole with the lysosomal marker Lamp-1 and diminished TCT entry (Romano et al., 2009).

## CONCLUDING REMARKS

What emerges from the data on signaling events during host cell invasion by *T. cruzi* is a still fragmentary picture. Although many parasite as well as host cell components have been identified as playing roles in MT or TCT invasion, these may represent only a small part of the repertoire available for the accomplishment of the critical step for infection. The whole process is beginning to be understood at the molecular level. Furthermore, how the diverse elements are connected and what are the sequences of reactions that culminate in intracellular rearrangements that facilitate parasite internalization have as yet to be clarified. Therefore, a formidable task is still ahead before we can more fully understand the intricate functioning of molecular and cellular machinery involved in *T. cruzi* invasion.

## Conflict of Interest Statement

The authors declare that the research was conducted in the absence of any commercial or financial relationships that could be construed as a potential conflict of interest.
